# Supplementary material for: Porcine Deltacoronavirus-like Particles Produced by a Single Recombinant Baculovirus Elicit Virus-Specific Immune Responses in Mice
Source: Viruses. 2023 Apr 29;15(5):1095. doi: 10.3390/v15051095 (PMC10221120; doi:10.3390/v15051095)
Supplement: Supplementary file 1 [file viruses-15-01095-s001.zip › viruses-2318775-supplementary.pdf]

Supplementary Material

Porcine deltacoronavirus-like particles produced by a single recombinant baculovirus elicit virus-specific immune responses in mice

Yangkun Liu[1†], Xueying Han[2†], Yaqi Qiao[3], Tiejun Wang[3], and Lunguang Yao[1*]

*** Correspondence:** Lunguang Yao: lunguangyao@163.com

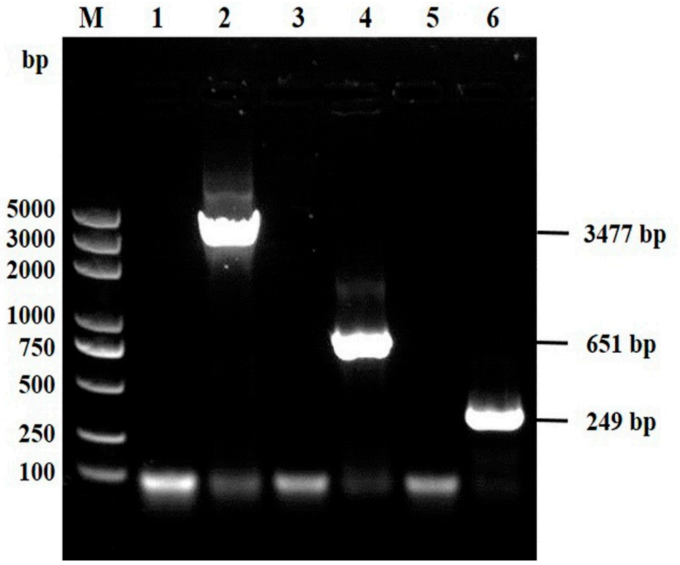

Supplementary Figure S1. PCR identification of the recombinant bacmids. Lane M: Trans2K Plus DNA Marker; Lane 1, 3 and 5: The PCR products obtained with AcMultiBac as negative control using primers SF and SR, MF and MR, EF and ER, respectively; Lane 2, 4 and 6: The PCR products obtained with rBacmid-MSE using primers SF and SR, MF and MR, EF and ER, respectively. The S, M and E fragments were approximately 3,477 bp, 651 bp and 249 bp, respectively.

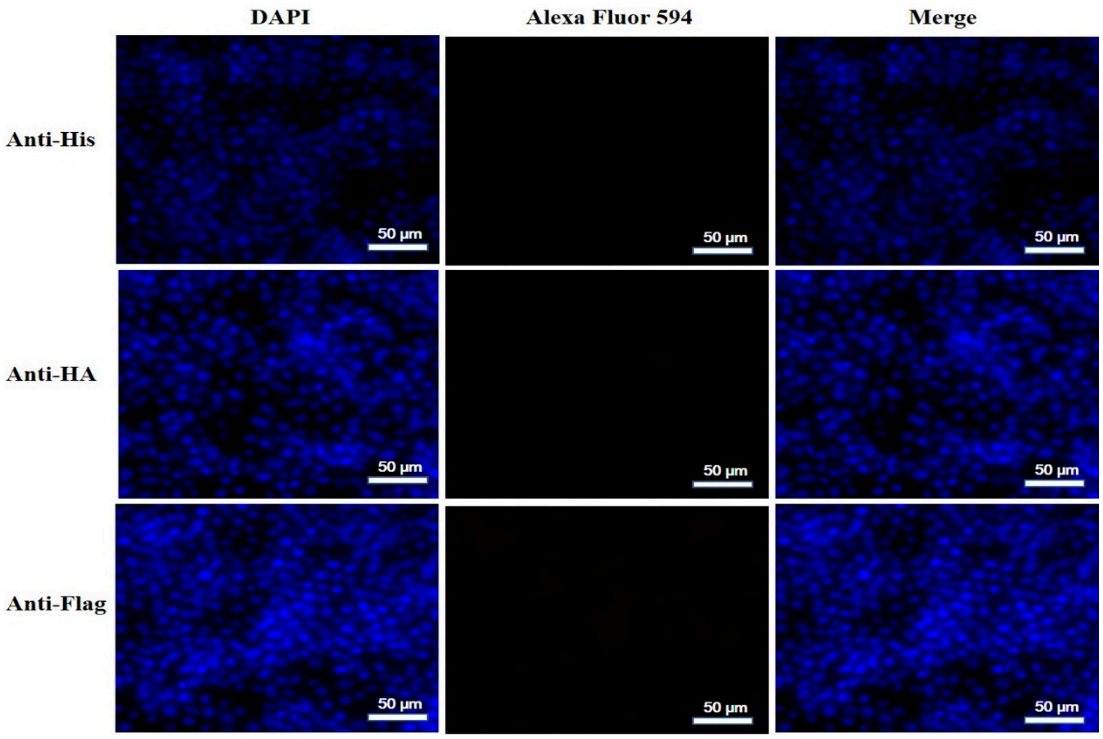

Supplementary Figure S2. Identification of the recombinant proteins expressed in normal Sf9 cells using IFA. Normal Sf9 cells were subjected to immunostaining using His, HA, and Flag antibodies. These cells were detected using fluorescence microscopy.
